# Germinal Center Dark Zone harbors ATR-dependent determinants of T-cell exclusion that are also identified in aggressive lymphoma

**DOI:** 10.21203/rs.3.rs-4093618/v1

**Published:** 2024-03-18

**Authors:** Valeria Cancila, Gaia Morello, Giorgio Bertolazzi, Allison Si-Yu Chan, Giulia Bastianello, Daniel Paysan, Patrick William Jaynes, Giovanna Schiavoni, Fabrizio Mattei, Silvia Piconese, Maria V Revuelta, Francesco Noto, Adele De Ninno, Ilenia Cammarata, Fabio Pagni, Saradha Venkatachalapathy, Sabina Sangaletti, Arianna Di Napoli, Davide Vacca, Silvia Lonardi, Luisa Lorenzi, Andrés J M Ferreri, Beatrice Belmonte, Gabriele Varano, Mario Paolo Colombo, Silvio Bicciato, Giorgio Inghirami, Leandro Cerchietti, Maurilio Ponzoni, Roberta Zappasodi, Fabio Facchetti, Marco Foiani, Stefano Casola, Anand D Jeyasekharan, Claudio Tripodo

**Affiliations:** 1 Tumor Immunology Unit, Department of Health Sciences, University of Palermo, Palermo, Italy; 2 Department of Economics, Business, and Statistics, University of Palermo, Palermo, Italy; 3 Cancer Science Institute of Singapore, National University of Singapore, Singapore; 4 IFOM ETS, The AIRC Institute of Molecular Oncology, Milan, Italy; 5 Laboratory for Nanoscale Biology, Paul Scherrer Institute, Villigen, Switzerland; 6 Department of Health Sciences and Technology, ETH Zurich, Zurich, Switzerland; 7 Department of Oncology and Molecular Medicine, Istituto Superiore di Sanità, Rome, Italy; 8 Department of Internal Clinical Sciences, Anesthesiology and Cardiovascular Sciences, Sapienza University of Rome, Rome, Italy; 9 IRCCS Fondazione Santa Lucia, Unità di Neuroimmunologia, Rome, Italy; 10 Laboratory affiliated to Istituto Pasteur Italia-Fondazione Cenci Bolognetti, Rome, Italy; 11 Division of Hematology and Oncology, Medicine Department, Weill Cornell Medicine and NewYork-Presbyterian Hospital, New York; 12 Institute for Photonics and Nanotechnologies, Italian National Research Council, Rome, Italy; 13 Department of Translational and Precision Medicine, Sapienza University of Rome, Rome, Italy; Neuroimmunology Unit, IRCCS Fondazione Santa Lucia, Rome, Italy; 14 Department of Medicine and Surgery, Pathology, IRCCS Fondazione San Gerardo dei Tintori, University of Milano-Bicocca, Italy; 15 Molecular Immunology Unit, Department of Experimental Oncology, Fondazione IRCCS Istituto Nazionale Tumori, Milan, Italy; 16 Pathology Unit, Department of Clinical and Molecular Medicine, Sant’Andrea University Hospital, Sapienza University of Rome, Rome, Italy; 17 Pathology Unit, ASST Spedali Civili di Brescia, Department of Molecular and Translational Medicine, University of Brescia, Brescia, Italy; 18 Lymphoma Unit, IRCCS San Raffaele Scientific Institute, Milan, Italy; 19 Vita-Salute San Raffaele University, Milan, Italy; 20 Department of Molecular Medicine, University of Padova, Padova, Italy; 21 Pathology and Laboratory Medicine Department, Weill Cornell Medicine and New York-Presbyterian Hospital, New York; 22 Pathology Unit, IRCCS San Raffaele Scientific Institute, Milan, Italy; 23 Department of Medicine, Weill Cornell, New York, NY, USA; 24 Department of Haematology-Oncology, National University Health System, Singapore, Singapore; 25 NUS Centre for Cancer Research, Yong Loo Lin School of Medicine, National University of Singapore, Singapore, Singapore; 26 Department of Medicine, Yong Loo Lin School of Medicine, National University of Singapore, Singapore, Singapore

## Abstract

The germinal center (GC) dark zone (DZ) and light zone (LZ) regions spatially separate expansion and diversification from selection of antigen-specific B-cells to ensure antibody affinity maturation and B cell memory. The DZ and LZ differ significantly in their immune composition despite the lack of a physical barrier, yet the determinants of this polarization are poorly understood. This study provides novel insights into signals controlling asymmetric T-cell distribution between DZ and LZ regions. We identify spatially-resolved DNA damage response and chromatin compaction molecular features that underlie DZ T-cell exclusion. The DZ spatial transcriptional signature linked to T-cell immune evasion clustered aggressive Diffuse Large B-cell Lymphomas (DLBCL) for differential T cell infiltration. We reveal the dependence of the DZ transcriptional core signature on the ATR kinase and dissect its role in restraining inflammatory responses contributing to establishing an immune-repulsive imprint in DLBCL. These insights may guide ATR-focused treatment strategies bolstering immunotherapy in tumors marked by DZ transcriptional and chromatin-associated features.

## INTRODUCTION

Tumor immune evasion is influenced by both tumor-cell-intrinsic and -extrinsic factors, including the silencing of tumor neoantigen presentation along with the capability to restrain immune cell infiltration and activation^1–2^. The variability in the composition of the ecosystems characterizing different tumors driven by diverse pathogenetic pathways is a major limitation to the identification of common determinants of T-cell-mediated response regulation and immune evasion. These mechanisms involve the establishment of inhibitory ligand/receptor checkpoint synapses^3^, generation of immunosuppressive environments by regulatory immune components such as regulatory T cells^4^ and suppressive myeloid elements^5^, and the refinement of the extracellular matrix meshwork toward immunomodulatory functions operated by structural mesenchymal elements and cancer cells that have undergone partial mesenchymal transition states^6–7^. Less characterized are mechanisms promoting exclusion or depletion of specific T-cell subsets. In epithelial cancers, T-cell exclusion is associated with tissue remodeling mediated by specialized fibroblast populations^8–9^, and programs involved in stroma remodeling and cell adhesion (e.g. WNT/b-catenin, TGFb, PI3K)^10–12^. However, the biological traits of tumors linked to T-cell depletion, particularly in histotypes like lymphomas, which typically lack distinct tumor/stroma boundaries, remain largely unexplored.

The germinal center (GC) is a highly intricate and dynamic microenvironment, wherein B cells responding to antigen undergo profound transcriptional and phenotypic changes, as a result of functional compartmentalization into dark zone (DZ) and light zone (LZ) areas^13–14^. The role of these specialized regions is to promote antigen-driven selection of particular B-cell clones, ensuring antibody affinity maturation and long-lived B cell memory^15^. Cell proliferation and immunoglobulin (Ig) somatic hypermutation confer to the GC DZ area a specialized function where tight coordination of antibody diversification and cell-cycle progression requires the establishment of a unique immunological niche^16^. Using spatially resolved approaches, we evaluated the in-situ microenvironment of the GC LZ and DZ, outlining their relationship with T-cell localization and phenotype. We identified the LZ/DZ interface as a barrierless constraint to intra-GC T-cell distribution and defined DZ-associated transcriptional and chromatin features negatively correlating with T-cell infiltration. The identification of a DZ-derived transcriptional core associated with T-cell exclusion in a subset of aggressive B-cell lymphomas underscores the exploitation of pre-existing immunological programs by immunologically ‘cold’ tumors. We highlight the importance of molecular determinants of DNA replication, damage response and repair regulated by the Ataxia Telangiectasia and Rad3 related (ATR) kinase, in restraining IFN signaling, maintaining the DZ transcriptional imprint and T-cell evasion.

## RESULTS

### The spatial transcriptome of the GC DZ is dominated by DNA replication and damage response

**Figure 1:**
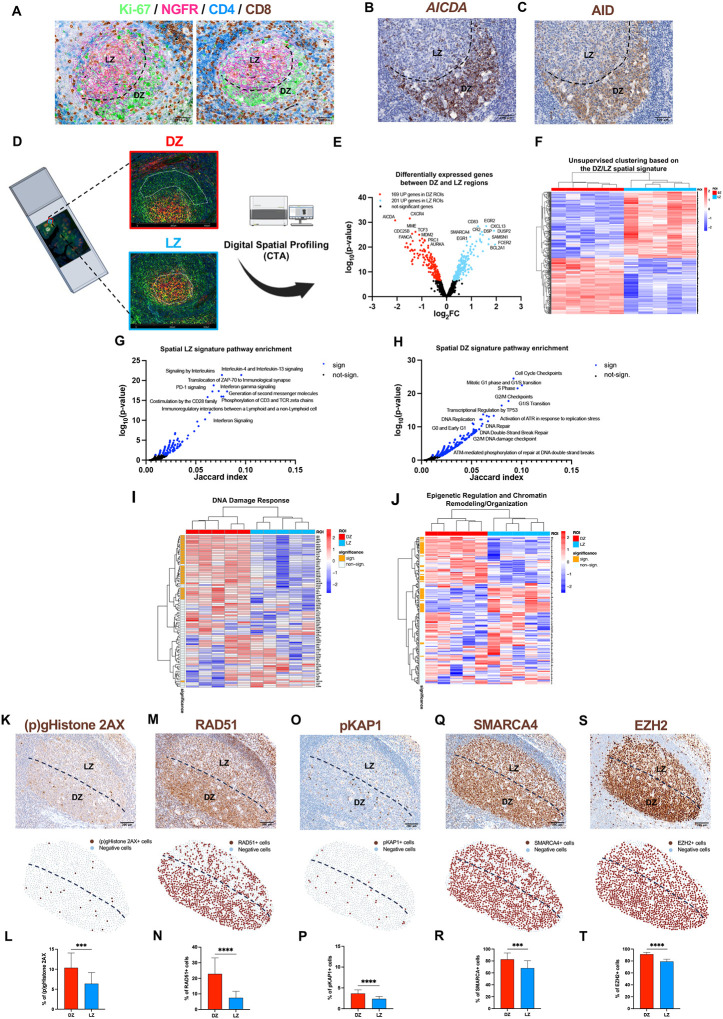
The GC DZ exhibits a spatial transcriptome primarily associated with DNA replication and damage response processes. **A,** Representative microphotographs of combined IHC/IF staining for Ki-67 (green signal), NGFR (pink signal), CD4 (blue signal), and CD8 (brown signal), showing dense expression of Ki-67 in GC DZ and NGFR expression in GC LZ regions. Original magnification, x200. Scale bar, 100 μm. **B-C,** Comparative images of mRNA *in situ* hybridization for *AICDA* and IHC for AID to evaluate the correspondence between mRNA and protein expression. Original magnification, x200. Scale bar, 100 μm. **D,** Digital spatial profiling experiment in DZ (*n* = 5) and LZ (*n* = 5) ROIs to identify an immune/stromal GC DZ/LZ signature. **E,** Volcano plot of differentially expressed genes (DEGs) from the comparison between DZ and LZ ROIs (adjusted p-values < 0.05). **F,** Heatmap of DEGs between DZ and LZ ROIs. The unsupervised hierarchical clustering based on the DZ/LZ spatial signature clearly discriminates DZ and LZ ROIs. **G-H,** Pathway enrichment of 201 LZ spatial signature genes and 169 DZ spatial signature genes (Reactome Pathway library). Significant pathways are marked with a blue colour. **I-J,** Expression of “DNA Damage Response” and “Epigenetic Regulation and Chromatin Remodeling/Organization” genes in DZ and LZ ROIs. The left bar indicates the significant DEGs between DZ and LZ ROIs (orange). **K-T,** Representative microphotographs, spatial plots and quantitative analyses of IHC for DNA damage/repair markers: (p)gHistone (**K** and **L**), RAD51 (**M** and **N**), pKAP1 (**O** and **P**), SMARCA4 (**Q** and **R**) and EZH2 (**S** and **T**) to assess the different enrichment between DZ and LZ (*n* GCs = 20). Original magnification, x100. Scale bar, 200 μm. Statistical analysis: two-tailed unpaired Mann-Whitney test (**L**, **N**, **P**, **R**, **T**). Mean ± standard error shown; *, P < 0.05; **, P < 0.01; ***, P < 0.001; ****, P < 0.0001.

In human tonsil, GCs exhibit a spatial compartmentalization featuring highly proliferative Ki-67-dense Dark Zone, and Follicular Dendritic Cell (FDC)-rich (NGFR+) Light Zone areas (Figure 1A). GCs are permeated by limited numbers of CD4+ and CD8+ T cells (Figure 1A). The DZ is highlighted by Activation Induced Cytidine Deaminase (AID) mRNA and protein expression (Figure 1B–C)^17^. To gain a comprehensive profiling of the immune and stromal components, we analyzed the *in situ* transcriptional profile of matched tonsillar GC DZ and LZ (n=10) regions of interest (ROIs) (Figure 1D) exploiting digital spatial profiling of 1824 curated genes^18–19^. We derived a DZ/LZ spatial signature of 370 differentially expressed genes (adj. p-value < 0.05), with 169 genes up-regulated in the DZ and 201 genes in the LZ (Figure 1E–F; Supplementary Table 1). The spatial LZ and DZ signatures were validated in GC B cell sc-RNAseq profiles^20^, where they efficiently discriminated DZ and LZ B cells (Supplementary Figure 1A-C). Analysis of transcripts discriminating DZ from LZ through both spatial and single cell expression profiling identified those predominantly modulated in the B cells of the two GC compartments (Supplementary Table 1). Spatial profiling confirmed hallmarks of DZ and LZ B-cell biology including *CXCR4*, *AICDA*, *CD27, TCL1A, AURKB, PLK1* (DZ markers) and *CD83*, *BCL2A1, CD40, IL21R, STAT6, EGR1* (LZ markers). Moreover, the spatial signatures unveiled differentially expressed microenvironment genes including the LZ-overexpressed transcripts *C3, CXCL14, MAF, IDO1, IL18, IL32*, and the DZ-associated transcripts *IL10RA, BMP7, LILRB2, SLAMF6*.

LZ and DZ molecular signatures showed different enrichment in molecular pathways (Figure 1G–H, Supplementary Table 2). DZ regions featured preferential expression of genes involved in the ATR-dependent DNA damage response pathway (Figure 1I; Supplementary Table 3), (*H2AX, BRCA1, PRKDC, RAD51)* and genes associated with epigenetic regulation and chromatin remodeling including *SMARCA4* and *EZH2* (Figure 1J; Supplementary Table 3), cell cycle checkpoints (Supplementary Figure 1D; Supplementary Table 3) and FOXO activation (Supplementary Figure 1E, Supplementary Table 3). In line with overexpression of DNA damage response transcriptional programs in the DZ, quantitative immunohistochemistry (IHC) for the DNA damage/repair marker phosphorylated (p)Histone 2AX S139 showed spatial enrichment within the DZ, and the same trend was observed for the DNA damage repair effector RAD51 and for the ATM substrate pKAP1 S824, marking DNA repair at heterochromatin^21^ (Figure 1K–P; Supplementary Figure 1F-H). Consistent with the transcriptional profiles, preferential expression of chromatin regulators SMARCA4 (BRG1) and EZH2 proteins, was detected in the DZ microenvironment (Figure 1Q–T; Supplementary Figure 1I-J).

### The GC DZ presents a microenvironment depleted of T-cell transcripts with a limited display of immune checkpoints

**Figure 2:**
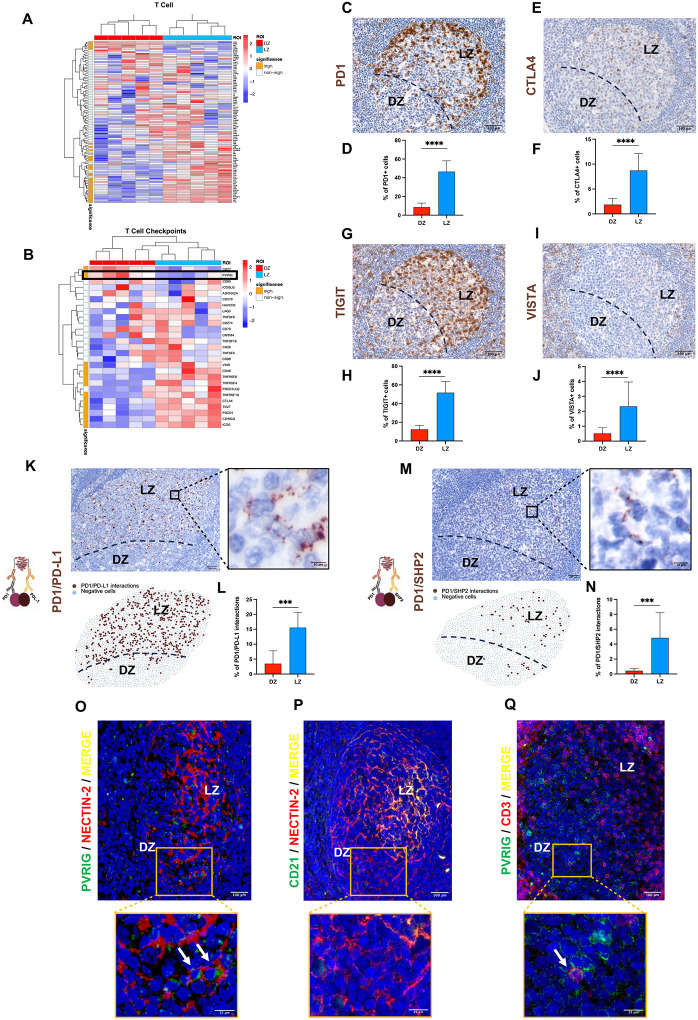
T-cell transcript depletion and limited immune checkpoint expression characterize the GC DZ microenvironment. **A-B,** Expression of “T-cell” and “T-cell checkpoints” genes in DZ and LZ ROIs. The left bar indicates the significant DEGs between DZ and LZ ROIs (orange). **C-J,** Representative microphotographs and quantitative analysis of IHC for PD1 (**C** and **D**), CTLA4 (**E** and **F**), TIGIT (**G** and **H**) and VISTA (**I** and **J**) showing a marked increase towards the LZ (*n* GCs = 20). Original magnification, x200. Scale bar, 100 μm. **K-N,** Representative microphotographs, spatial plots and quantitative analyses showing PD1/PD-L1 (**K** and **L**) or PD1/SHP2 (**M** and **N**) interactions (brown signal) detected by in situ proximity ligation assay (*n* GCs = 10). Original magnifications, x100 and x630 (insets). Scale bars, 200 μm and 10 μm. **O,** Double-marker IF of PVRIG (green signal) and NECTIN-2 (red signal) showing the association in the DZ GC. **P,** Double-marker immunofluorescence of CD21 (green signal) and NECTIN-2+ (red signal) highlighting NECTIN-2 expression beyond the LZ pattern. **Q,** Double-marker IF of PVRIG (green signal) and CD3 (red signal) showing scattered double positive T cells infiltrating the DZ. Original magnifications (**O**, **P**, **Q**), x200 and x400 (insets). Scale bars, 100 μm and 25 μm. Statistical analysis: two-tailed unpaired Mann-Whitney test (**D**, **F**, **H**, **J**, **L, N**). Mean ± standard error shown; *, P < 0.05; **, P < 0.01; ***, P < 0.001; ****, P < 0.0001.

Mutagenic and DNA damaging microenvironments, such as the GC DZ, represent potential immune-activating settings^22^ implying either effective checkpoint control over immune cell activation, or immune exclusion. Based on the spatial profiling, the DNA damaging environment of the DZ demonstrated a significant under-representation of T-cell-associated transcripts (Figure 2A; Supplementary Table 3). Transcripts encoding pivotal immune checkpoints regulating T-cell function, such as *PDCD1, CTLA4, TIGIT*, and *VSIR*, showed a markedly decreased expression in the DZ (Figure 2B; Supplementary Table 3), consistent with their protein expression being spatially biased towards the LZ (as shown in Figure 2C–J). Moreover, the actual engagement of the prototypical immune checkpoint receptor/ligand pair PD1/PD-L1, visualized through *in situ* proximity ligation assay (PLA), proved to be nearly absent in the GC DZ and limited to sparse elements in the LZ (Figure 2K–L). *In situ* PLA also revealed PD1/SHP2 interactions restricted to the LZ confirming local activation of the PD1 inhibitory signaling (Figure 2M–N).

Spatial profiling identified the CD8+ T-cell/NK-associated *PVRIG* receptor as the only inhibitory immune checkpoint upregulated in the DZ (Figure 2B). Double-marker immunofluorescence (IF) analyses confirmed PVRIG (CD112R) protein expression in the GC DZ and highlighted PVRIG+ cells in contact with stromal elements expressing the PVRIG-ligand NECTIN-2 (CD112) (Figure 2O). Indeed, NECTIN-2+ stromal cells extended beyond the LZ CD21+ FDC meshwork, within the DZ (Figure 2P). A fraction of the scattered PVRIG+ elements infiltrating the DZ were identified as T cells according to CD3 co-expression (Figure 2Q). Taken together, these results underline that T-cell inhibitory checkpoints expressed in the GC are under-represented and not engaged in the DZ, except for the PVRIG/NECTIN-2 axis.

### A repulsive pattern is observed for T cells in the DZ, involving IFNG+ cells marginalized at the LZ/DZ interface

**Figure 3:**
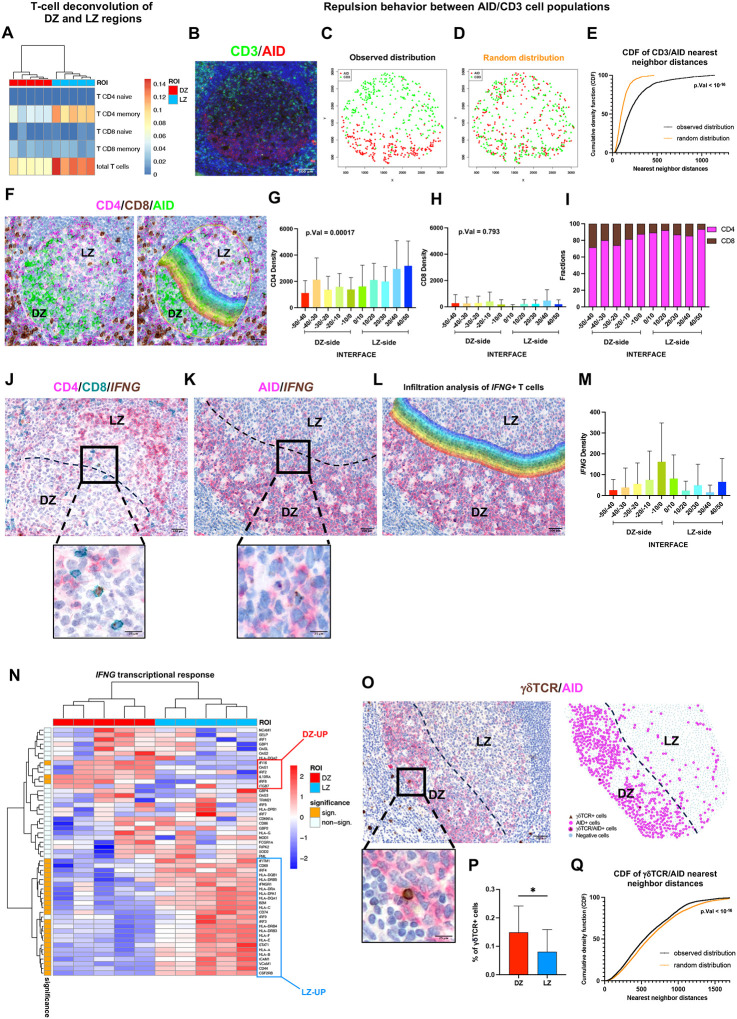
T-cell repulsion and IFNG+ cell marginalization at the LZ/DZ interface in the GC microenvironment **A,** SpatialDecon T-cell fractions over DZ and LZ ROIs. **B,** Representative microphotographs of double-marker IF for CD3 (green signal) and AID (red signal) within the GC. Original magnification, x200. Scale bar, 100 μm. **C-D** Example of observed (**C**) and randomized (**D**) spatial distribution. **E,** Cumulative density functions (CDFs) of CD3-AID nearest neighbor distances calculated in the observed samples (black curve) and in the randomized samples (orange curve). The distances between CD3 and AID cells are significantly higher in the observed samples compared with the randomized samples (Wilcoxon p-value < 10^−16^), indicating a segregation among the two cell populations. **F,** Representative microphotographs of triple immunohistochemical staining for CD4 (pink signal) CD8 (brown signal) and AID (green signal) (left) and DZ/LZ infiltration analysis representation (right) to quantify the density of CD4+ and CD8+ T cells infiltrating the interface within the GC. Original magnification, x200. Scale bar, 100 μm. **G-H,** Average density of CD4+ (**G**) and CD8+ (**H**) cells along with the LZ/DZ transition (*n* GCs = 10). **I,** Average fraction of CD4 and CD8 positive cells along the interface (*n* GCs = 10). **J,** Representative microphotographs of combined mRNA *in situ* hybridization of *IFNG* (brown signal) and double-marker immunohistochemistry of CD4 (pink signal) and CD8 (green signal). Original magnification, x200 and x400 (insets). Scale bars, 100 μm and 25 μm. **K-M,** *In situ* detection for *IFNG* mRNA and IHC for AID representative images (**K**), DZ/LZ infiltration analysis representation (**L**) and quantitative analyses of the average density of *IFNG+* cells infiltrating the inside and outside of the interface (**M**). (*n* GCs = 10). Original magnification, x200 and x400 (insets). Scale bars, 100 μm and 25 μm. **N**, Expression of IFNG transcriptional response genes in DZ and LZ ROIs. The left bar indicates the significant DEGs between DZ and LZ ROIs (orange). **O-P,** Representative microphotographs, spatial plots and quantitative analyses of IHC for δTCR cells show different spatial enrichment and expression in DZ and LZ (*n* GCs = 20). Original magnification, x100 and x400 (insets). Scale bars, 200 μm and 25 μm. **Q,** CDFs of AID-δTCR nearest neighbor distances calculated in the observed samples (black curve) and the randomized samples (orange curve). The population distances are significantly lower in the observed samples compared with the randomized samples (Wilcoxon p-value < 10^−16^). It indicates an aggregation behavior among the two cell populations. Statistical analysis: two-tailed unpaired Mann-Whitney test (**P**). Mean ± standard error shown; *, P < 0.05; **, P < 0.01; ***, P < 0.001; ****, P < 0.0001.

We then assessed T cell enumeration in the DZ and LZ through spatial transcriptional deconvolution^23^. A significantly lower fraction of T cell subsets was estimated in the DZ areas as compared with the corresponding LZ (Figure 3A). To quantitatively evaluate the exclusion of T cells from the GC DZ, we applied an *ad-hoc* developed algorithm^24^ to spatial maps of GC CD3+ T cells and AID+ DZ cells revealed by double-marker IF. Spatial analysis of CD3+ and AID+ cell distribution highlighted that these cell populations reciprocally diverged, indicating a repulsive pattern (Figure 3B–E). We observed that immune exclusion from the DZ also involved Foxp3+ regulatory cells (Supplementary Figure 2A-C) and sparsely distributed CD3-CD57+ NK cells populating the GC (Supplementary Figure 2D-F). The exclusion did not extend to CD68+ macrophages, which were similarly distributed in the LZ and DZ (Supplementary Figure 2G-I; Supplementary Table 3).

T cells in the GC are predominantly CD4+ T helper cells, with a minor component of CD8+ T cells^25^. We investigated whether the exclusion of T cells from the DZ was similar for CD4+ and CD8+ GC T cells. Using spatial maps of triple IHC for CD4 CD8 and AID, we delineated a 100μm-wide LZ/DZ interface (centered on the edge of AID+ cells) and quantified the density of CD4+ and CD8+ T cells infiltrating the inner (DZ side) and outer (LZ side) layers of the interface (Figure 3F). The analysis revealed different infiltration profiles of CD4+ and CD8+ T cells, since the density of CD4+ T cells progressively dropped along with the LZ/DZ transition, while the amount of CD8+ T cells remained steadily low at both sides of the interface (Figure 3G–I).

Using a nearest neighbor spatial analysis, we investigated the interaction between CD4+ and CD8+ T cells with DZ and LZ B cells, which were identified based on the IHC expression of the differential markers PLK1 (DZ B cells) (Supplementary Figure 3A-B) and EGR1 (LZ B-cell cells) (Supplementary Figure 3C-D). Both CD4+ and CD8+ T cell populations exhibited a preference for interacting with LZ B cells and CD8+ T cells exhibited an overall closer proximity to DZ cells compared to CD4+ T cells (Supplementary Figure 3A-D).

The interactions of T cells within the GC are primarily finalized to provide B-cell help supporting antibody affinity maturation and competitive selection via costimulatory and cytokine signals delivery^26^, while the role of effector T cells is less characterized. We investigated whether a fraction of GC T cells with effector phenotype could be identified *in situ*, using combined CD4 and CD8 double-marker IHC and Interferon gamma (*IFNG)* mRNA ISH. *IFNG*+ T cells were detected within the GC, preferentially characterizing a subset of CD8+ T cells (Figure 3J). A comparative analysis of the frequency of *IFNG+* CD4+ and CD8 + T cells in GC areas and in T-cell-rich peri-follicular regions revealed that despite the total number of CD4+ and CD8+ T cells was substantially higher in the peri-follicular regions, the relative fraction of *IFNG*+ T cells was significantly enriched within the GC microenvironment (Supplementary Figure 4A-D). *IFNG*-expressing elements in the GC showed preferential localization outside the DZ, at the DZ/LZ interface (Figure 3K–M), which suggested that their immune function could be associated to the interaction with B-cell mutants exiting the DZ. Accordingly, an *IFNG* transcriptional signature differentially characterized LZ and DZ regions (Figure 3N; Supplementary Table 3).

Among non-Tfh T-cell subsets, we also explored the presence of gamma-delta T (γδT) cells^27^ and their distribution within the GC. Very few γδT cells were detected in the GCs through TCRdelta quantitative IHC (Figure 3O). These sparse γδT cells populating the GC were preferentially localized within the DZ microenvironment (Figure 3P–Q), highlighting γδTCRexpressing cells as outliers in DZ T-cell repulsive pattern.

These findings indicate that the complex organization of T-cell subsets in the GC is influenced by a phase separation between the LZ and the DZ in the absence of a physical barrier, which reflects on the polarized distribution of T cells, including *IFNG*+ CD8 effectors.

### The DZ and LZ microenvironments are defined by gradients in chromatin compaction

**Figure 4:**
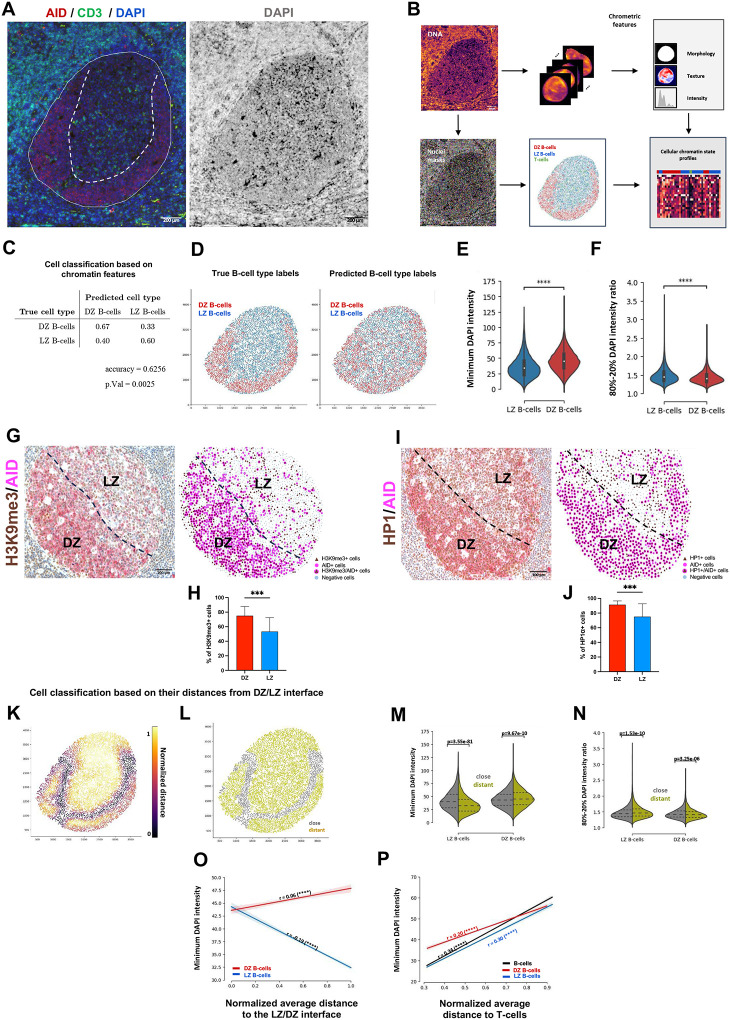
Chromatin compaction gradients differentiate the DZ and LZ microenvironments. **A,** Representative microphotographs of a GC showing the AID (red signal) and CD3 (green signal) staining (left) and the DAPI (DNA), marked in white (right). Original magnification x100. Scale bar, 200 μm. **B,** Overview of the computational pipeline to characterize the cell-type identities and chromatin states of cells from input fluorescent images. **C,** Average of the row-normalized confusion matrices of the RFC trained to distinguish between LZ and DZ B-cells. The average is obtained by evaluating the RFC in a 10-fold stratified cross-validation setup for a balanced random subsample of DZ and LZ B-cells (n=9,197). The prediction accuracy (Acc = 0.635) is significantly higher than the No Information Rate (NIR = 0.5, p-value 0.0025, one-sided Wilcoxon signed-rank test). **D,** Visualization of the prediction performance of the RFC for a GC sample. The true cell-type labels are shown on the left. Cell type labels predicted by an RFC when holding out the respective nuclei during training of the RFC are shown on the right. **E-F,** Violin plots showing the distribution of the “minimum DNA intensity” and the “ratio of the 80-to-20 percentile of the DNA intensity” among the LZ/DZ B-cell populations (Welch’s t-test, p-value < 1e-124). **G-J,** Representative microphotographs, spatial plots and quantitative analyses of double-marker IHC for AID (DZ marker) and H3K9me3 (**G** and **H**) or HP1 (**I** and **J**) to assess the different enrichment between DZ and LZ (*n* GCs = 20). Original magnification, x100. Scale bar, 200 μm. Statistical analysis: two-tailed unpaired Mann-Whitney test (**H** and **J**). Mean ± standard error shown; *, P < 0.05; **, P < 0.01; ***, P < 0.001; ****, P < 0.0001. **K,** Identified distance to the DZ/LZ interface of the individual cells by their corresponding color coding. **L,** Binary classification of cells close (grey) or distant (olive) to the interface by thresholding the distance measure at 0.4. **M-N,** Violin plots showing the distribution of the “minimal DNA intensity” and the “ratio of the 80 and 20 percentiles of the DNA intensity distribution” for LZ and DZ B-cells in close proximity (grey) and those distant (olive) to the LZ/DZ interface. The means are found to differ significantly (Welch t-test). The inner dashed lines correspond to the 25, 50 and 75 percentiles. **O,** Visualization of the significant correlation of the minimum DNA intensity of LZ/DZ B-cells with respect to their range-normalized distance to the LZ/DZ interface (Pearson r=0.0671 and r=−0.1902, p-values < 1e-6, permutation test). Linear regression lines with corresponding 95% bootstrapping confidence intervals using b=1,000 bootstrap samples are shown as shaded regions. **P,** Visualization of the significant correlation of the minimum DNA intensity of all B-cells (black), DZ (red) and LZ B-cells and their average range-normalized distance to T-cells in the germinal centers (Pearson r=0.3410, r=0.2030 and r=0.2998, p-values < 1e-6, permutation test). Linear regression lines with corresponding 95% confidence intervals using b=1,000 bootstrap samples are shown as shaded regions.

We investigated whether nuclear chromatin organization characteristics concur with the phase separation between cells in the DZ and LZ, in light of the differences between these regions with regard to the spatial profiles of genes involved in DNA replication and damage response, chromatin organization and remodeling. Using DAPI-stained nuclei from GC IF images, we extracted *chrometric* features to analyze nuclear morphology and chromatin organization (see Methods). A random forest model, trained with AID/CD3/DAPI IF data, effectively classified DZ and LZ B cells (Figure 4A–C) and the resulting predictions recapitulated the spatial organization of the two regions (Figure 4D). The key discriminators were features related to chromatin compaction (Supplementary Table 4), with DZ B-cells showing increased compaction (Figure 4E–F). These results highlight that the transcriptionally distinct DZ and LZ regions display nuclei with different mechanical properties resulting from different chromatin compaction states. This was confirmed by higher levels of heterochromatin-associated Histone H3K9me3 (Figure 4G–H), Heterochromatin Protein 1 (HP1) (Figure 4I–J) and EZH2 in DZ areas (Figure 1S–T).

We subsequently investigated whether the DZ/LZ interface could be identified as the topographic determinant in the transition between DZ and LZ chromatin compaction states. By measuring segmented cells’ distances from this interface (Figure 4K, see Supplementary Methods) and analyzing *chrometric* features, we found distinct chromatin states, particularly in LZ cells near the interface, which displayed a more DZ-like phenotype (Figure 4L; Supplementary Table 4). Chromatin compaction inversely correlated with LZ B cells distance from the interface, and a similar trend was observed in DZ cells (Figure 4M–O). Additionally, a positive correlation between chromatin compaction and distance from T-cells was observed in both DZ and LZ cells (Figure 4P). These results indicate that in the GC microenvironment, the localization and chrometric states of B cells are linked and give rise to discrete gradients correlating with DZ/LZ compartmentalization and T-cell segregation.

### The cGAS-STING pathway is inactive in the T-cell depleted DZ microenvironment

**Figure 5:**
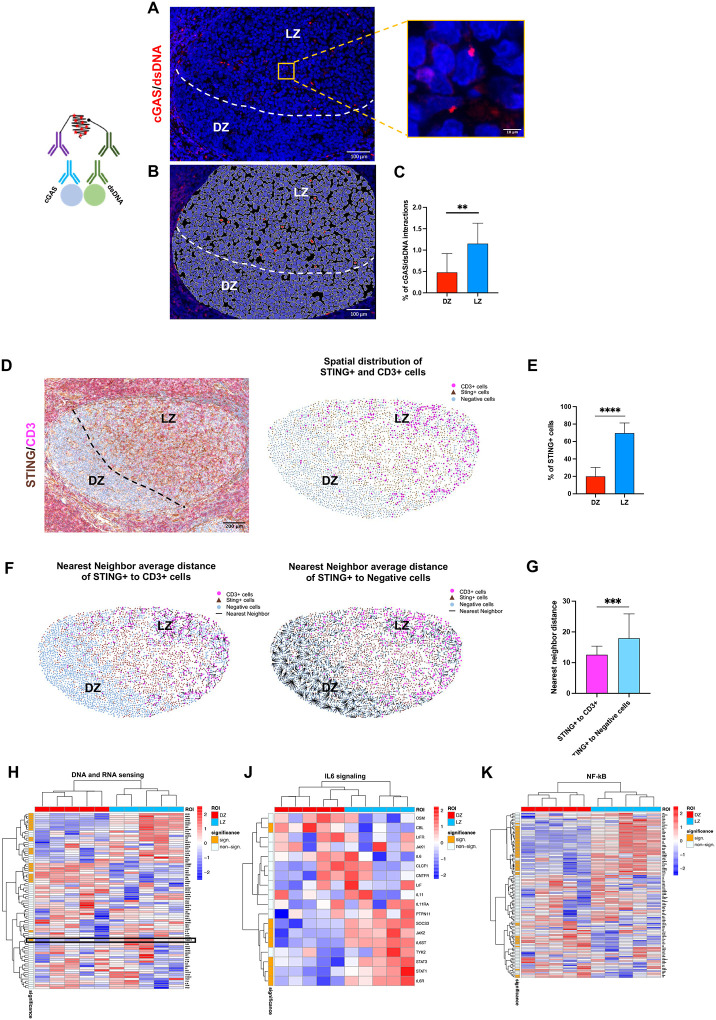
The T-cell depleted DZ microenvironment exhibits inactivity of the cGAS-STING pathway. **A-C,** Representative microphotographs, spatial plots and quantitative analyses showing cGAS/dsDNA interactions (red signal) detected by fluorescent *in situ* proximity ligation assay (*n* GCs = 10) and showing scattered elements in the LZ regions. Original magnification, x200 and x630 (insets). Scale bars, 100 μm and 10 μm. **D-E,** Representative microphotographs, spatial plots and quantitative analyses of STING (brown signal) and CD3 (pink signal) double-marker immunostaining highlighting a different spatial distribution of STING and CD3 in DZ and LZ (*n* GCs = 20). Original magnification, x100. Scale bar, 200 μm. **F-G,** Nearest neighbor distance of STING to CD3 and STING to Negative cells showing the proximity of STING to CD3 cells (*n* GCs = 20). Statistical analysis: two-tailed unpaired Mann-Whitney test (**C, E, G**). Mean ± standard error shown; *, P < 0.05; **, P < 0.01; ***, P < 0.001; ****, P < 0.0001. **H-K,** Expression of “DNA and RNA sensing” (**H**), “IL6 signaling” (**J**), and “NF-kB” (**K**) genes in DZ and LZ ROIs. The left bar indicates the significant DEGs between DZ and LZ ROIs (orange).

Along with activation of the DNA damage response, DZ B cells exhibited higher chromatin compaction. This characteristic was linked to reduced presence of T cells and diminished expression of immune checkpoints in the DZ. Consequently, the DZ appears as a “cold” environment, purportedly less permissive than the LZ for triggering inflammatory responses associated to release in the cytosol of double-stranded (ds)DNA. To test this hypothesis, we performed an *in situ* PLA experiment to detect in GCs the direct engagement of the cGAS cytoplasmatic sensor by dsDNA. PLA detected focal interaction events within the GCs and these events were predominantly localized in the LZ (Figure 5 A–C), with almost no reactivity in the DZ, indicating that events enabling cGAS engagement by cytoplasmic dsDNA can occur in the GC but were precluded in the DZ microenvironment. Further supporting LZ-associated cGAS activation, expression of the cGAS inflammatory pathway effector *TMEM173* (STING) was preferentially detected in LZ B cells (Figure 5D–E), marking close spatial proximity to T cells (Figure 5F–G). Consistently, molecular pathways associated with DNA and RNA sensing and involved in immune activation were mostly overexpressed in the LZ as compared with the neighboring DZ regions (Figure 5H–K; Supplementary Table 3).

### DZ spatial signature negatively correlates with T-cell infiltration in DLBCL

T cell exclusion from the DZ linked to the absence of inflammatory signaling and silencing of immune checkpoints, despite strong activation of DNA damage response pathways, suggests the existence of local negative determinants of T-cell infiltration and activation. We hypothesized that similar mechanisms may be co-opted in malignancy. To test this hypothesis, we focused on Diffuse Large B-cell Lymphomas (DLBCL), a heterogenous set of aggressive neoplasms which encompasses the whole spectrum of lymphomagenesis in the GC^28–30^.

**Figure 6:**
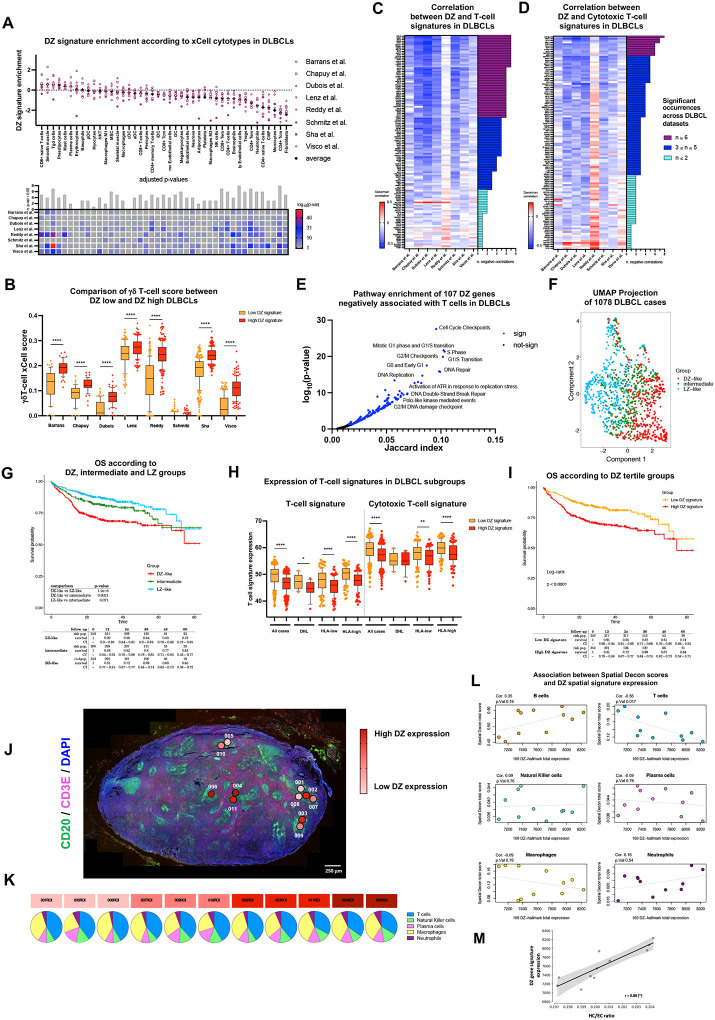
The GC DZ spatial signature in aggressive B cell lymphomas is associated with reduced T cell infiltration. **A,** DZ enrichment scores indicate the association between DZ gene expression and xCell cytotype scores calculated in 8 DLBCL datasets. Positive DZ enrichment values indicate a positive association between the DZ spatial signature and the xCell cytotype scores, while negative values indicate a negative association. The bottom panel highlights the significance of the enrichment scores (Wilcoxon adjusted p-values). **B,** Comparison of γδT-cells xCell score between low DZ expression and high DZ expression DLBCL cases. DZ high and DZ low groups have been obtained classifying the DLBCL cases based on the tertile separation of the DZ total expression. Wilcoxon p-values have been calculated to compare the xCell scores among the DZ high and the DZ low groups (*, P < 0.05; **, P < 0.01; ***, P < 0.001; ****, P < 0.0001). **C-D,** Observed correlations between the DZ spatial signature genes and the average expression of the T-cell hallmark/Cytotoxic T-cell gene signature in 8 DLBCL datasets. The right bars indicate how many times a gene was found to be significantly correlated over the DLBCL datasets. Violet bars indicate genes significantly correlated with the T cell signature in at least six DLBCL datasets. Blue and light-blue bars indicate genes significantly correlated less than six times. **E,** Pathway enrichment (Reactome Pathway library) of 107 DZ genes negatively correlated with the T-cell signatures in at least 3 DLBCL datasets. Significant pathways are marked with a blue color. **F,** UMAP projection of 1078 harmonized DLBCL cases classified based on the DZ/LZ spatial signature. DZ-like cases (red), LZ-like cases (light blue), and intermediate cases (green) are highlighted in the UMAP. **G,** Overall survival over DZ-like, LZ-like, and intermediate patients from the harmonized dataset (1078 cases). **H,** Expression of T-cell signatures over DLBCL patient subgroups. The DLBCL subgroups refer to double-hit lymphoma cases (DHL), high HLA expression (HLA-high), and low HLA expression (HLA-low) cases. Wilcoxon p-values have been calculated to compare the T-cell gene expression between DZ high expression and DZ low expression patients. **I,** Overall survival over high DZ expression and low DZ expression groups from the 1078 harmonized DLBCL cases. **J,** Digital spatial profiling experiment in 11 ROIs selected within CD20+ (green signal) and CD3E (red signal) infiltrates of a lymph node involved by diffuse large B-cell lymphoma (DLBC). Original magnification, x50. Scale bar, 250 μm. **K-L,** Association between the DZ spatial signature expression and SpatialDecon cytotype scores over 11 IG ROIs, reporting the Kendal correlation coefficient and p-values. **M,** Scatterplot shows the measured DZ gene signature expression of the ROIs (n=11) plotted against the median heterochromatin-to-euchromatin (HC/EC) ratio of the nuclei in those regions. The black line shows the fit of a linear regression model which visualises the significant correlation of the two quantities (Pearson r=0.8843, p-value = 0.0180, permutation test). A 95% confidence interval computed using 1,000 bootstrap samples for the regression line is shown as the shaded region in grey.

We interrogated the transcriptomes of 3610 DLBCL cases from 8 independent reference cohorts for expression of the DZ spatial signature^31–38^. We investigated the relationship of the DZ signature with the immune and stromal composition according to xCell transcriptional deconvolution algorithm^39^. DLBCL cases stratified based on DZ signature enrichment showed significant differences in terms of immune/stromal microenvironment composition (Figure 6A; Supplementary Table 5). Notably, DZ-like cases displayed lower frequencies of most T cell populations, except for γδT-cells (Figure 6B), in line with our finding from reactive GCs.

We further analyzed the correlation between the expression of each of the 169 DZ-associated transcripts with the expression of T-cell and Cytotoxic T-cell hallmark gene signatures over the different DLBCL cohorts (Supplementary Figure 5A-B; Supplementary Table 6). A significant negative correlation with T cell signatures was identified for 107 DZ signature genes in at least three different DLBCL datasets (Figure 6C–D; Supplementary Figure 6A-B; Supplementary Table 6), indicating that a DZ spatial signature enriched in ATR-dependent DNA damage response and cell cycle checkpoints programs was negatively associated with T-cell content in DLBCL (Figure 6E; Supplementary Table 6).

To evaluate the effect of the DZ or LZ spatial signature enrichment on prognosis, we focused on the transcriptomes of 1078 aggressive B-cell lymphomas including DLBCL and high-grade B-cell lymphomas harmonized from of two clinically-annotated RNA-seq-profiled datasets (GSE117556; GSE32918). Cases clustered according to the expression of DZ and LZ spatial signatures (DZ-like, LZ-like and Intermediate, Figure 6F). DZ-like and LZ-like cases showed significantly different prognostic behaviors (Figure 6G) with DZ-like cases showing worse Overall Survival (OS). The performance of the DZ spatial signature alone (not in combination with LZ spatial signature) was also analyzed confirming that cases with higher DZ signature had lower expression of T-cell signatures (Figure 6H; Supplementary Table 7) and highlighting that the same cases were characterized by a shorter OS (Figure 6I). In these cases, the unfavorable influence of DZ spatial signature on OS emerged also when cases with germinal center-related (GCB-) and activated B-cell type (ABC-) cell of origin were separately analyzed (Supplementary Figure 6C-D).

The composition of the tumor microenvironment in DLBCL has been correlated with MHC expression status^40^ and mutations involving HLA genes. Impaired MHC expression associates with DLBCL enriched with gene expression characteristic of *MYC/BCL2* double-hit biology^40^, which overlapping strongly with dark zone biology. According to our spatial transcriptional profiling, the expression of MHC class-I/-II genes was significantly lower in DZ regions when compared to LZ regions for most HLA gene transcripts with the exception of *HLA-G*, -*DQA2*, and -*DPB1* (Supplementary Figure 6E; Supplementary Table 3). We therefore investigated whether the negative correlation of the DZ spatial signature with T-cell infiltration in DLBCL was related to their MHC gene expression. The 1078 cases were split based on the expression of MHC class-I/-II genes (Supplementary Table 7). In both the HLA-high and HLA-low groups, higher expression of the DZ signature associated with decreased T cell signatures expression (Figure 6H; Supplementary Table 7), indicating that the DZ spatial signature negatively correlated with T cell infiltration beyond MHC gene expression status. We further tested this hypothesis in the setting of MYC/BCL2 double-hit lymphomas (DHL), a highly aggressive form of GC-derived B-cell lymphoma that we and others have previously linked with a DZ-like profile^41–42, 20^ and that has been reported as generally characterized by an immune depleted microenvironment^43^. Among the 35 DHL cases analyzed, those displaying a high DZ signature showed lower expression of the T-cell signature (Figure 6H). These results demonstrate that the GC DZ spatial signature is able to trace a DZ-like biology in aggressive B cell lymphomas that involves attenuated T cell infiltration.

We further investigated if similar patterns of T-cell depletion, indicative of a DZ transcriptional imprint, were present intralesionally. Utilizing digital spatial profiling, we analyzed the transcriptomes of 11 regions of interest (ROIs) within CD20+ infiltrates in a lymph node sample from a case of non-GC DLBCL, as classified by the Hans algorithm^44^, exhibiting MYC and BCL2 double-expression (Figure 6J; Supplementary Table 8). The ROIs were sorted based on their DZ spatial signature expression levels, and their T-cell content was estimated by transcriptional deconvolution. In the ROIs, DZ signature expression levels inversely correlated with T-cell infiltration (Figure 6K–L), whereas no significant correlation was found with other microenvironment constituents, like macrophages (Figure 6K–L). Reduced T-cell presence in microregions with higher DZ signature expression was confirmed by CD20 and CD3 IF (Supplementary Figure 6F). In this setting we further investigated the relationship between DZ spatial signature expression in the DLBCL ROI and nuclear chromatin compaction. Applying the same nuclear segmentation and *chrometric* features extraction methodology used in reactive GC DZ and LZ analyses, a positive correlation between the median heterochromatin-to-euchromatin content ratio in cells from the 11 DLBCL ROI and their respective DZ signature expression emerged (Figure 6M). This finding aligns the local transcriptional activation of DZ genes with increased nuclear chromatin compaction.

### Depletion of native immune and stromal components through DLBCL xenografting enforces a DZ-like transcriptional imprint

**Figure 7:**
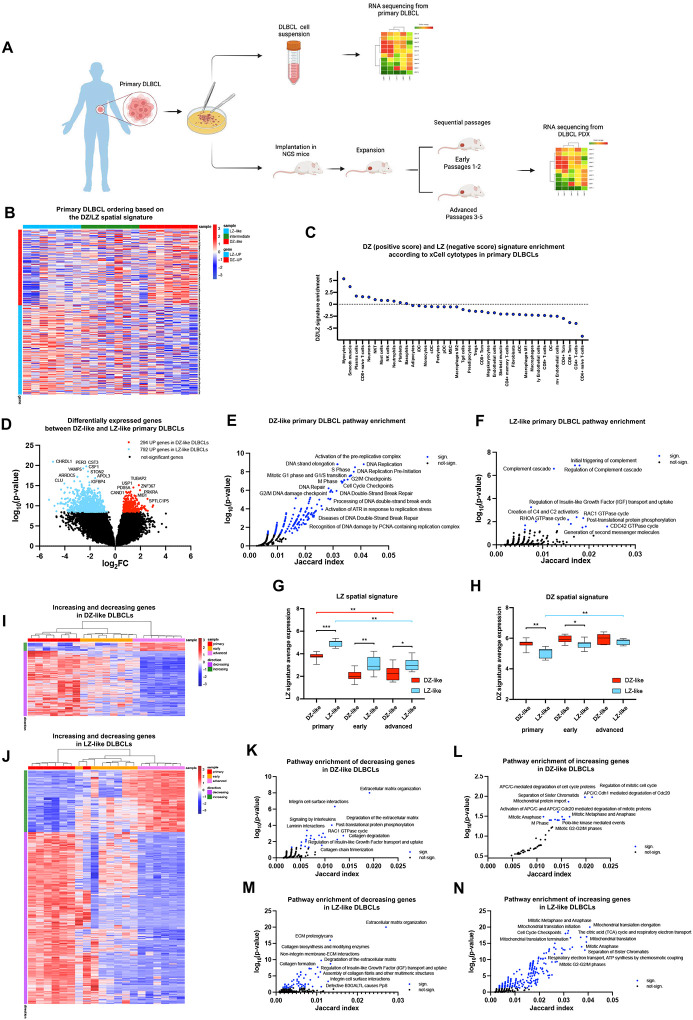
The absence of a native microenvironment attenuates the DZ-like/LZ-like DLBCL divergence in PDXs **A,** Graphical scheme of RNA-seq transcriptomes analyzed from 21 primary DLBCL tumors and the corresponding patient-derived xenografts (PDXs). **B,** Heatmap of primary DLBCL samples categorized in DZ-like, LZ-like, and intermediate based on the DZ/LZ spatial gene expression signature**. C,** Enrichment scores indicate the association between DZ/LZ gene expression and xCell cytotype scores calculated in 8 DLBCL datasets. Positive values indicate cytotypes that enrich the DZ-like primary DLBCLs, while negative values indicate cytotypes that enrich the LZ-like primary DLBCLs. **D,** Volcano plot of differentially expressed genes (DEGs) from the comparison between DZ-like and LZ-like primary DLBCL samples (adjusted p-values < 0.05, abs-logFC>0.58). **E-F,** Pathway enrichment of 294 genes UP in DZ-like DLBCLs and 792 genes UP in LZ-like DLBCLs (Reactome Pathway library). Significant pathways are marked with a blue colour. **G-H,** Average expression of DZ/LZ spatial signature among primary, early, and advanced DLBCLs. Wilcoxon test was used for pairwise comparisons between DZ-like and LZ-like samples. The Kruskal-Wallis test was used to compare three groups (red and light-blue lines indicate KW test significance. *, P < 0.05; **, P < 0.01; ***, P < 0.001; ****, P < 0.0001). **I-J,** Heatmap of increasing and decreasing genes in primary, early, and advanced DLBCLs. Significant genes were selected based on the non-parametric one-way ANOVA and log-FCs (Kruskal-Wallis adj. p-value < 0.05, pairwise log-FCs > 0.58). **K-N,** Pathway enrichment of significant decreasing and increasing genes among primary, early, and intermediate DLBCLs in DZ-like and LZ-like subgroups.

To explore the functional association between the DZ and LZ spatial signatures in DLBCL and the immune and stromal microenvironment, we analyzed RNA-seq transcriptomes from 21 primary DLBCL tumors and their corresponding patient-derived xenografts (PDX) at early (passages 1–2) and advanced (passages 3–5) time points (GSE145043) (Figure 7A). Based on the expression of DZ and LZ spatial signatures, the primary DLBCL tumors were categorized as DZ-like, LZ-like, or Intermediate (Figure 7B). The proportions of immune and stromal populations in DZ-like (n=7) and LZ-like (n=7) tumors, estimated using transcriptional deconvolution, were coherent with their respective DZ-like and LZ-like profiles, with DZ-like cases showing significantly lower fractions of several T-cell populations (Figure 7C). DZ-like and LZ-like DLBCL exhibited differential expression of 1086 genes (Figure 7D; Supplementary Table 9). These genes were consistently enriched in molecular processes such as DNA replication, DNA damage repair, ATR response to replicative stress (overexpressed in DZ-like), TCR/ZAP70 signaling, complement activation, and RHOA/RAC GTPase activity (overexpressed in LZ-like) (Figure 7E–F; Supplementary Table 9). However, upon analyzing the transcriptomes of the corresponding PDX, the differential expression of genes, which served as distinguishing features for DZ-like and LZ-like cases, drastically dropped (Supplementary Figure 7A). This emphasizes that the distinct biological features observed in primary tumor transcriptomes converged towards less diverse biologies in the absence of a native microenvironment. Indeed, the expression of the LZ spatial signature, indicative of LZ-like cases, progressively decreased at early and advanced PDX time points (Figure 7G; Supplementary Table 10) due to the gradual depletion of transcripts associated with T-cells (*CD3D/E, CD4, CD8, TRBC1, CTLA4, ICOS, FOXP3*), follicular dendritic cells (*CLU, VCAM1*), extracellular matrix and stromatogenesis (*SPARC, FN1, LAMA1, LAMB1, COL1A2, COL3A1, COL4A1, COL5A2, COL6A3*), as well as the reduced expression of pro-inflammatory genes (*TMEM173/STING1, IL1B, IL18, IL33*). When the expression of a B-cell associated LZ signature (Supplementary Table 1) was analyzed, the difference between LZ-like and DZ-like cases maintained a consistent level across primary tumors, early and advanced PDX (Supplementary Figure 7B). In contrast, the same LZ-like cases exhibited a progressive increase in the expression of the DZ spatial signature across early and advanced PDX (Figure 7H). The analysis of a B-cell DZ gene signature yielded similar results, with LZ-like cases showing increased expression of DZ B-cell genes in early and advanced PDX (Supplementary Figure 7C). This gain was aligned with the increasing expression of transcripts related to cell cycle and DNA replication (*E2F1, CCNB1, CCNB2, FOXM1, PLK1, CDC20, AURKB*), ATR-dependent response to replication stress (*RPA2, RFC2, CDK2, CDC25C*) and DNA repair (*H2AX, RAD51, POLE3*) (Supplementary Table 10). Along with these genes, other genes characterizing DZ biology, such as the transcription factor *TCF3*, the B-cell receptor component *CD79B*, and the polyamine metabolism regulator *OAZ1*^41^ were progressively induced (Supplementary Table 10), further substantiating the enforced DZ transcriptional imprint. The transcriptional alterations observed in DZ-like cases from primary tumors to early and advanced PDX were much less pronounced than those observed in LZ-like cases (Figure 7I–J). They involved a progressive decline in the expression of genes implicated in extracellular matrix and vascular stromatogenesis (*COL4A1, COL4A2, COL6A1, VWF, KDR, THBS1*), along with an increase in the expression of DZ cell cycle genes (*FOXM1, PLK1, CDC20, AURKB*) and genes involved in tricarboxylic acid cycle respiration (*CS, COX5A, MDH2, MT-ATP8*) (Figure 7K–N; Supplementary Table 10).

We also investigated whether DZ-like and LZ-like lymphomas xenografted into immunocompromised mice exhibited distinct stromal and/or innate immune responses, as inferred from the analysis of murine transcripts. Using molecular deconvolution^45^ and single-cell RNA seq datasets (i.e. Tabula Muris compendium profiles of immune and stromal cells represented in NSG hosts such as monocytes, macrophages, dendritic cells, granulocytes, stromal cells, and endothelial cells)^46^ we did not observe significant differences in the expression of mouse (host-derived) transcripts in the PDX from DZ-like and LZ-like cases indicating a similar stromal host response (Supplementary Figure 7D).

These results imply that the depletion of immune and stromal components of the lymphoma microenvironment that results from xenografting into immune-compromised mice attenuates the DZ-like/LZ-like DLBCL divergence.

### DZ spatial signature is independent of Aid-driven mutagenesis

**Figure 8:**
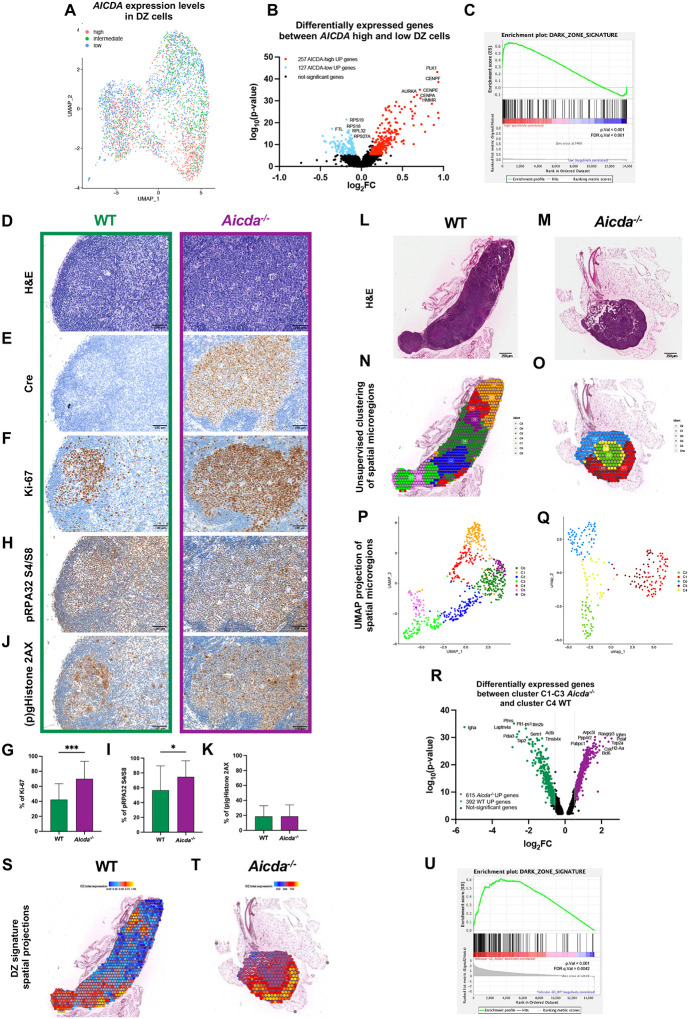
The spatial signature of DZ cells is independent of AICDA-related mutational processes. **A,** UMAP projection of 4.082 cells from the Holmes et al. dataset. The cells are classified as low, intermediate, and high AICDA gene expression. While low indicates the absence of expression, and high indicates an expression greater than the 2nd tertile. **B,** DEGs from the comparison between AICDA-high and AICDA-low cells from the Holms et al. single-cell dataset (Wilcoxon Rank Sum test adj. p-value < 0.05, abs-logFC > 0.25). **C,** GSEA enrichment analysis on AICDA-high and AICDA-low cells. The DZ spatial signature strongly enriches AICDA-high cells in the Holmes et al. dataset (p-value < 0.001). **D-K,** Comparative microphotographs of H&E (**D**) and IHC for Cre (**E**), Ki-67 (**F** and **G**), pRPA32 S4/S8 (**H** and **I**) and (p)gHistone2AX (**J** and **K**) in mesenteric lymph nodes of WT and *Aicda*^*−/−*^ mice. Ki-67 (**G**), pRPA32 S4/S8 (**I**) and (p)gHistone2AX (**K**) show different expression between WT and *Aicda*^*−/−*^ mice (*n* GCs = 20). Original magnification, x200. Scale bar, 100 μm. **L-M,** Representative microphotographs of H&E-stained sections from WT and *Aicda*^*−/−*^ mesenteric lymph node involved in the Visium spatial transcriptome experiment profiling. Original magnification, x50. Scale bar, 250 μm. **N-O,** Unsupervised clustering of spatial microregions. **P-Q,** UMAP projection of the spatial microregions. Colors reflect the unsupervised cluster classification. **R,** DEGs from the comparison between the WT cluster 4 and *Aicda*^*−/−*^ clusters 1 and 3 (Wilcoxon Rank Sum test adj. p-values < 0.05, abs-logFC > 0.025). **S-T,** Spatial projection of the DZ spatial signature total expression in WT and *Aicda*^*−/−*^ samples. **U,** GSEA enrichment analysis on follicular-GC microregions. The spatial DZ spatial signature significantly enriches follicular-GC regions of the *Aicda*^*−/−*^ sample (p-value < 0.001).

DNA replication checkpoint and DNA damage response transcriptional programs consistently emerged as determinants of the DZ spatial signature we found negatively associated with T-cell infiltration in GCs and DLBCL. As a preeminent mediator of the DZ B cell mutator profile, AID promotes DNA mutagenesis and repair during Ig SHM, also playing a central role in the regulation of epigenetic heterogeneity of GC B-cells^47^. We analyzed the expression of the DZ spatial signature in the transcriptomes of DZ B cells classified according to *AICDA* expression status, exploring the association between *AICDA* and elements of the DZ signature at the single cell level. scRNA-seq-profiled purified DZ cells (GSE139891) were classified as *AICDA*-high (*AICDA* expression > Tertile 2) and *AICDA*-low (absence of detectable *AICDA* expression) (Figure 8A) and the differential gene expression profile was analyzed. The differentially expressed genes (abs. logFC > 0.25, adj. p-value < 0.05) consisted of 384 genes, 257 of which were significantly overexpressed in *AICDA*-high and 127 in *AICDA*-low DZ cells (Figure 8B; Supplementary Table 11). The transcriptomes of *AICDA*-high DZ cells were significantly enriched in genes involved in DNA replication and cell cycle checkpoints including *PLK1, CCNB2, CDC20* (Supplementary Figure 8A; Supplementary Table 11), while *AICDA*-low cells were marked by genes involved in transcriptional regulation and in nucleotide mismatch repair, such as *POU2F2, FEN1 and UNG* (Supplementary Figure 8B; Supplementary Table 11). A significantly higher expression and positive enrichment of the DZ spatial signature was observed in *AICDA*-high as compared with *AICDA*-low DZ cells (Figure 8C; Supplementary Table 12), indicating that the DZ spatial signature primarily characterizes DZ cells marked by elevated *AICDA* expression.

To weigh the biological relevance of Aid-associated mutagenesis for the establishment/maintenance of the DZ transcriptional signature, we analyzed *Aicda* deficient (*Aicda*^tm1(cre)Mnz/J^) mice^48^. The GCs spontaneously forming in the mesenteric lymph nodes (MLNs) of *Aicda*^*−/−*^mice homogeneously expressed Cre, were larger than the WT counterpart (Figure 8D–E), showed a higher Ki-67+ proliferative fraction compared to WT controls, higher expression of pRPA32 S4/S8 and comparable frequencies of (p)gHistone2AX (Figure 8F–K) implying enhanced replicative potential and higher replicative stress in Aid-deficient GCs. We performed a spatial transcriptome experiment profiling 1950 microregions from two MLNs of WT (1270 microregions) and *Aicda*^*−/−*^ (680 microregions) genotype. Unsupervised clustering of the spatial transcriptomes from the MLNs identified 7 microregion clusters in WT MLN and 5 clusters *Aicda*^*−/−*^ MLN (Figure 8L–Q). The 7 WT microregion clusters included one cluster formed by follicular/GC microregions (C4), four clusters within para-cortical regions variably characterized by T cell- and macrophage-related transcripts (C0, C2, C3, C5), and two additional clusters in the medullary regions enriched in plasma cells, macrophages/dendritic cells, endothelial and mesenchymal cells (C6, C1) (Supplementary Table 13). In Aid−/− mutant mice, two clusters were identified in follicular/GC regions (C1, C3), two clusters in paracortical areas enriched in T cells and macrophages (C2, C4), and one cluster relative to medullary areas enriched in macrophage and endothelial/mesenchymal cell transcripts (C0) (Supplementary Table 13). We compared the transcriptional profiles of the spatial regions of WT and *Aicda*^*−/−*^ MLNs corresponding to follicle GC microregions (WT C4 and *Aicda*^*−/−*^ C1+C3 clusters, Supplementary Figure 9A-B) and identified 1007 differentially expressed genes (392 upregulated in WT and 615 upregulated in *Aicda*^*−/−*^, Figure 8R; Supplementary Figure 9C-D; Supplementary Table 14). Among the top differentially expressed transcripts were Ig heavy chain constant region transcripts *Igha* and *Ighm* that were upregulated in WT and *Aicda*^*−/−*^ respectively, consistent with the inability of *Aicda*^−/−^ GC B cells to undergo IgH isotype switching, and with the predominant switching to IgA in MLN B cells of WT mice. *Aicda*^−/−^ follicular/GC microregions were characterized by the upregulation of transcripts associated with the DZ spatial signature, which included genes involved in transcriptional and epigenetic regulation (*E2f1, Bcl6, Pou2af1, Ezh2, Dnmt1, H3f3a, Crebbp, Smarca4*), DNA replication and repair (*Top2a, Pold4, Brca1, Rad51, Rad21,Msh6*, *Neil1, Stmn1*), B-cell receptor signaling (*Cd79b, Syk*), and chemotaxis (*Cxcr4*). The DZ spatial signature was globally enriched in *Aicda*^−/−^ follicular/GC microregions as compared with WT ones (Figure 8S–T), indicating that in the absence of Aid activity, DZ transcriptional programs result from enhanced replicative potential and resulting replication stress (Figure 8U; Supplementary Table 12). DZ signature enrichment in *Aicda*^−/−^ was also confirmed on bulk RNA-seq of three WT and three *Aicda*^−/−^ MLN samples (Supplementary Figure 9E; Supplementary Table 12) that comprised the samples profiled by spatial transcriptomics.

Among genes differentially modulated in the follicular/GC microregions in the absence of Aid were also genes related with MHC presentation. Indeed, class-I MHC genes including *H2-d1, H2-k1*, and *B2m*, were significantly downregulated in comparison to Aid-proficient microregions (Supplementary Table 14), in line with the DZ transcriptional imprint associating with dampened antigen presentation programs. These results indicate that the DZ spatial signature correlates with *AICDA* expression in DZ cells, yet it primarily reflects the GC B cells’ response to DNA replication, replicative stress, and subsequent repair activities, being, *de facto*, independent of B-cell specific Aid-mediated mutational processes for its induction and maintenance.

### ATR inhibition in DZ-like DLBCL cells dampens the DZ transcriptional imprint and immune exclusion

ATR-dependent DNA damage sensing, response and repair pathways consistently emerged from transcriptional profiling of the DZ microenvironment. Additionally, these pathways positively enriched the DZ spatial signature negatively associated with T-cell infiltration in DLBCL. ATR kinase is required to protect cells from replicative stress and was shown to behave like a sensor of mechanical stress at the nuclear envelope^49^ preventing nuclear collapse and NE ruptures and consequent activation of the inflammatory cGAS/STING^50–51^. The DZ is characterized by high proliferation rate and increased chromatin compaction. High levels of ATR activity in the DZ might be therefore required to cope with mechanical stress and replication stress and contribute to prevent cGAS-STING activation.

**Figure 9:**
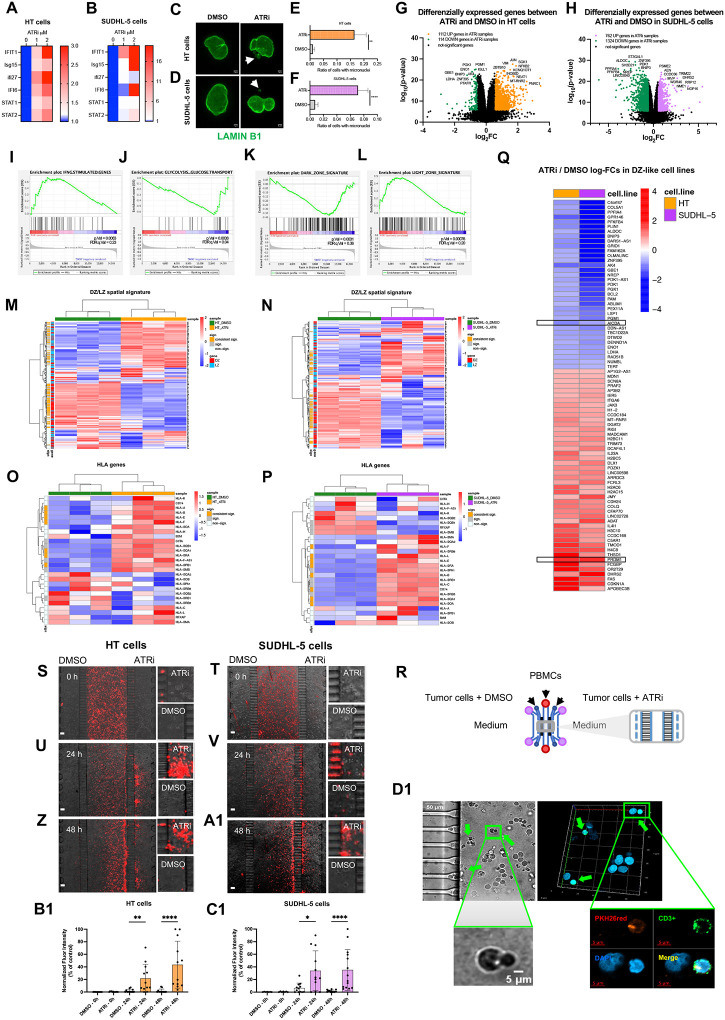
ATRi unleashes immune permeation of a DZ-like DLBCL milieu in a competitive on chip assay **A-B,** qPCR analysis showing Interferon-Stimulated Genes (IFNG) induction in HT (**A**) or SUDHL-5 (**B**) cells following a 48h treatment with AZD6738 at the indicated concentrations. **C-D,** Representative immunofluorescence images showing micronuclei formation in HT (**B**) and SUDHL-5 (**C**) cells treated with 1μM ATR inhibitor for 48h (green: laminB1 staining decorating the nuclear envelope)**. E-F,** Micronuclei quantifications (relative to IF analysis **C-D**) showing an increased ratio of micro-nucleated cells in the samples treated with 1μM ATRi for 48h (**E**: HT cells, **F**: SUDHL-5 cells). **G-H**, Differentially expressed genes from the comparison between ATRi and DMSO samples in HT/SUDHL-5 cell line (adjusted p-value < 0.05, |log-FC|>0.58). **I-L,** GSEA enrichment analysis on ATRi and DMSO samples. The IFNG Stimulated pathway (**I**) and the LZ spatial signature (**L**) significantly enrich the ATRi samples. The Glycolysis Glucose Transport pathway (**J**) and the DZ spatial signature (**K**) significantly enrich the DMSO samples **M-P,** Expression of DZ/LZ spatial signature (**M** and **N**) and HLA genes (**O** and **P**) in HT and SUDHL-5 cell lines. The left bar indicates the significant DEGs between ATRi and DMSO. The orange colour indicates the significant DEGs whose FCs have a consistent value among cell lines. **Q,** log-FC values from the comparison between ATRi vs DMSO in DZ-like cell lines (i.e., HT and SUDHL-5) considering only the significant genes shared between both cell lines. Positive log-FC values indicate genes up-modulated by ATRi (red cells in the heatmap), while negative log-FC values indicate genes down-modulated by ATRi (blue cells in the heatmap). **R**, Schematic representation of the competitive device. PKH26-labeled PBMCs were loaded in the central fluidic chamber. DLBCL (HT or SUDHL-5) cells were embedded in Matrigel with ATRi or DMSO and loaded in lateral chambers. **S-T,** Distribution of red fluorescent PBMCs after cell loading. **U-A1** Preferential migration of PBMCs towards lateral DLBCL-gel chambers after 24h **(U** and **V**) and 48h **(Z** and **A1)** from cell loading. **B1-C1,** Quantitative analysis of PBMC infiltration expressed by integrated density of red fluorescence in the two HT **(B1)** or SUDHL-5 **(C1)** Matrigel chambers. Mean of representative fields ± S.D. from 3 replicates of different donor PBMCs (n=3) is shown. **D1,** Confocal analysis of PKH26+ CD3+ T cells in the ATRi-treated DLBCL-gel chamber (48h time point) showing close interaction with DLBCL cells. Lower left panel visible light image depicting a tumor cell interacting with an infiltrated T cell inside the Matrigel chamber. Green box shows a magnification of a T lymphocyte interacting with a tumor cell. Right panel, Z stack acquisition from the panel J with a magnification (green box) displaying the strict spatial interaction between CD3+ PKH26+ T cells and DAPI+ DLBLCL (HT) cells. The green box delineates a representative Z stack plan evidencing a T lymphocyte interacting with a DLBCL cancer cell. Images were acquired at the 48h time point. Statistical analysis: two-tailed unpaired Mann-Whitney test (**E, F, B1, C1**). Mean ± standard error shown; *, P < 0.05; **, P < 0.01; ***, P < 0.001; ****, P < 0.0001.

On these bases, we functionally investigated whether ATR inhibition (ATRi) in lymphoma cells with a DZ-like transcriptional profile, could perturb the immunologically-cold status activating the expression of genes associated with inflammatory signaling, such as IFN-stimulated genes. Two DLBCL cell lines, HT and SUDHL-5, were selected according to their elevated expression of the DZ spatial signature (Supplementary Figure 10A). The cells were treated with either the clinical-grade ATRi Ceralasertib (AZD6738) (1 or 2 micromolar) or the ATRi solvent DMSO (as control) for 48h. ATRi induced the expression of several IFN-stimulated genes (*ISG15, IFIT1, IFI6, IFI27, STAT1, STAT2, STAT3)* (Figure 9A–B). Moreover, ATRi treatment significantly increased the formation of micronuclei, structures highly prone to NE ruptures known to activate the cGAS-STING pathway^52^ (Figure 9C–F). These results indicated that ATR interference was sufficient to flare genome instability-associated inflammatory signaling in DZ-like lymphoma cells.

To gain a comprehensive insight into the modifications induced by ATRi on DZ-like DLBCL cell gene expression, we analyzed by RNA-seq the whole transcriptome of HT and SUDHL-5 DZ-like DLBCL cells following 48h treatment with ATRi (1 micromolar) or DMSO. In this time window, ATRi treatment did not affect the viability of HT and SUDHL-5 cells (Supplementary Figure 10B-C). ATRi induced significant transcriptional changes in HT and SUDHL-5 (Figure 9G–H, Supplementary Table 15) leading to positive enrichment of IFN-stimulated genes in both cell lines (Figure 9I; Supplementary Table 12). Additionally, ATRi resulted in the negative enrichment of genes associated with glycolysis and glucose transport (Figure 9J; Supplementary Table 12). The ATRi-induced transcriptional reprogramming of DLBCL cell implied the negative regulation of the DZ spatial signature genes and, conversely, the induction of LZ signature transcripts (Figure 9K–N, Supplementary Tables 12 and 16). The transcriptional rewiring imposed by ATRi led to an increase in the levels of MHC-I and -II transcripts in treated DZ-like DLBCL cells, supporting the reversal of their immune-evasive profile (Figure 9O–P, Supplementary Table 16).

Such DZ-to-LZ transcriptional modulation was marked by the overexpression of the *PRDM1* gene, which encodes for BLIMP-1, a key transcription factor responsible for terminating the GC program and initiating plasma cell differentiation, and by the consistent downregulation of the DZ hallmark *AICDA* (Figure 9Q; Supplementary Table 15). To investigate whether the transcriptional modifications induced by ATRi in DZ-like DLBCL cells could eventually impact on their immune repulsive behavior, we adopted a competitive microfluidic assay^53–54^. An *ad hoc* fabricated device composed by three main fluidic chambers and two Matrigel-containing chambers interconnected by two arrays of microchannels, was used to co-culture peripheral blood mononuclear cells (PBMCs) with HT or SUDHL-5 DZ-like DLBCL cells, allowing the comparison of two different treatment conditions of lymphoma cells simultaneously (Figure 9R). Lymphoma cells were embedded in Matrigel in the presence of ATRi (1 micromolar) or DMSO, and loaded into opposite lateral chambers, while PBMCs were loaded in the central fluidic chamber. Prior to their introduction into the device, PBMCs were marked with the red fluorescent cell tracker PKH26, allowing for the quantitative analysis of their migration towards the DLBCL cells treated with ATRi or DMSO, at various intervals, using fluorescence microscopy. At the beginning of the experiments (0h), the PBMCs were uniformly distributed into the central chamber (Figure 9S–T). After 24h and 48h culture, the PBMCs permeated the chambers containing ATRi-treated HT (Figure 9U, Z) or SUDHL-5 (Figure 9V, A1) cells, while remaining repelled from DMSO-treated cells. A significant infiltration of the PBMCs inside the ATRi-treated DLBCL Matrigel chambers was scored at 24h and 48h time points as compared with DMSO-treated DLBCL chambers (Figure 9B1–C1). Among infiltrating PBMCs a fraction of PKH26+CD3+ T-cells was detected, which showed direct spatial interaction with PKH26− DLBCL cells (Figure 9D1). The results from this competitive microfluidic assay indicate that ATR inhibition is effective in unleashing immune attraction towards DZ-like DLBCL cells.

## DISCUSSION

The DZ, a hub for B cell proliferation and Ig hypermutation, is marked by a fine equilibrium between genomic stability and immune surveillance, actively regulating its microenvironment and affecting overall immune response. This regulation is reflected in the DZ’s transcriptional core, which is rich in DNA replication checkpoints and ATR-dependent DNA damage response modulators. DZ spatial signature genes are linked with the high levels of DNA damage inherent to the proliferation and mutational processes of the GC^55^. Our transcriptome experiments in *Aicda*^*−/−*^ mouse lymph nodes point to DNA replicative stress as the primary driver of DZ spatial transcriptional programs^56^ encompassing S-phase-associated ATR-dependent DNA damage response genes. Thus, the upregulation of such DZ transcriptional core genes serves as a protective mechanism ensuring genomic stability in highly proliferating B cells. Our findings suggest that this transcriptional adaptation to replicative stress may also help prevent T cell entry into the DZ during B cell clonal expansion and IgV gene diversification. Although representing a minor fraction, IFNG-producing CD8+ T cells emerge from our *in situ* analyses as a GC-resident subset mainly found at the LZ/DZ border, excluded from the DZ, indicating a niche for potential effector cells. DZ-associated low MHC-I/-II expression would suggest a bias towards DZ immune surveillance by TCR-independent immune cells, like NK cells. However, CD57+CD3− NK elements were not exempt from substantial exclusion from the DZ. We identified PVRIG as the only T/NK immune checkpoint to be overexpressed in the DZ. Given our observation of rare CD8+ elements percolating in the DZ and the finding of the rare GC-infiltrating γδT-cells preferentially residing in the DZ, we can envisage a function for PVRIG in controlling the activation of the rare T and NK cells that succeed to infiltrate the DZ. Through the interaction with Nectin-2 ligand expressed by FDCs and DZ stromal cells, PVRIG could exert its co-inhibitory function reported for NK and effector T cells^57^. The DZ spatial signature includes transcripts like *PVRIG* and *BMP7*, directly linked to the suppression of T-cell activation and infiltration. BMP7 has been shown to limit T-cell infiltration and reduce the effectiveness of immune checkpoint inhibitors in breast cancer models^58^. The DZ spatial transcriptome presents a novel perspective on the role of DNA replication and damage response genes in immune regulation indicating that its core genes are involved in maintaining genomic stability, but also in shaping the immune microenvironment. Our analysis of DLBCL PDXs indicates that the gradual reduction of native immune and stromal elements enforces DZ transcriptional imprint. These changes may be associated with events favoring the selection of DNA repair pathways, particularly those involved in intra-S and S-G2 repair, contributing to the tumor’s adaptive growth capabilities. Regulation of chromatin compaction emerged among the DZ spatial signature programs. Chromatin condensation distinguishes DZ and LZ cell populations and may be tied to their cell cycle distribution. This disparity likely arises from the unique DNA repair demands in DZ cells, driven by S-phase replication and G1-linked AID mutagenesis in the DZ, alongside with chromatin condensation during mitosis. Intermediate chromatin states at the DZ-LZ interface hint at cell cycle exit facilitating chromatin relaxation. Chromatin compaction serves dual functions: safeguarding DNA in swiftly dividing B cells from damage, and regulating gene expression related to B cell maturation, GC program cessation, DNA damage response, and immune activity. Our results suggest that chromatin compaction could contribute to the exclusion of T cells by both giving rise to a barrierless DZ/LZ separation and regulating genes involved in T-cell signaling and distribution. Evidence includes chromatin compaction gradients meeting at the LZ/DZ boundary and DZ spatial signatures aligning with overexpression of heterochromatic markers such as pKap1 S824, EZH2, H3K9me3, and HP1. We demonstrate that the negative influence of DZ transcriptional identity over T-cell distribution and content extends beyond the GC LZ/DZ functional compartments, involving DLBCL. In that setting, analysis of DZ upregulated genes, positive hallmarks of the DZ spatial signature, indicate a direct influence of DZ molecular programs over T-cell infiltration. From the analysis of transcriptomic data of eight independent cohorts of DLBCL, higher expression of the DZ spatial signature associated with lower estimated fractions of T-cell subsets including CD4+ and CD8+ T central and effector memory. An opposite trend was noted for γδT-cell estimated fractions that resulted significantly higher in cases with higher DZ spatial signature expression. This finding follows up on the recent demonstration of γδT-cells playing a major role as effectors of anti-tumor T-cell responses in MHC-low cancers^59^. Indeed, the DZ is an MHC-I/-II-low environment; moreover, aggressive B-cell lymphomas with DZ-like transcriptional imprints display reduced MHC-I/-II gene expression and include MYC/BCL2 DH high-grade B-cell lymphomas displaying frequent mutations in MHC genes^42^.

The finding that ATR-related pathways are central elements of the transcriptional core derived from the DZ underscores the importance of ATR in defining the distinct features of the DZ. In a previous report, it was proposed that BCL6 transcriptionally represses ATR in purified centroblasts^60^. Our profiling of DZ and LZ native environments did not reflect these results, suggesting that BCL6 repression of ATR transcription may be dynamic and influenced by tissue-level B/T interactions, which differently characterize the DZ/LZ dichotomy. It is plausible that GC B cells or their transformed counterparts can manipulate ATR-dependent DNA damage response genes to regulate immune surveillance. These genes could contribute to immune evasion by promoting genomic stability and restraining signals that could activate the immune system, such as cGAS/STING engagement. Clinical-grade ATR inhibitor experiments support this hypothesis, showing ATRi’s effect on IFN-stimulated genes in two DZ-like DLBCL cell lines, alongside with negative modulation of DZ spatial signature genes. ATRi significantly upregulated PRDM1 in these cell lines, linking ATR response to GC DZ transcriptional identity maintenance. In the setting of B-cell lymphomas, the expression of a DZ-like transcriptional profile has been associated with highly aggressive diseases including Burkitt and DH lymphomas^61,41^. The latter subset represents an unmet therapeutic challenge due to the failure of conventional chemo-immunotherapy^62^. We underline here that treatments based on the exploitation of anti-tumor immune effectors either through the re-activation of checkpoint-inhibited TILs or the transfer of enhanced effectors (e.g. chimeric antigen receptor T-cells) could be underpowered when in the presence of an elevated DZ spatial signature expression.

By dampening DZ signature expression and through the flaring of inflammatory signaling and MHC genes upregulation, ATRi could represent a promising novel strategy for enhancing T-cell permeation and activation. On this same ground, overexpression of the nuclear pore component XPO1 has been suggested to compensate in aggressive B-cell lymphomas for MYC-induced replication stress through the induction of key replication checkpoints listed among our DZ spatial signature hallmarks such as RAD51, WEE1, and BRCA1^62^. XPO1 inhibition therefore represents a promising complementary target to inhibit replication stress-associated DZ signature limiting immune activation^63^. Using a competitive microfluidic assay, we show that ATRi treatment reverts immune cell exclusion by DZ-like DLBCL cells. Although we identified a new level of regulation of molecular programs associated with DZ biology by ATR, the precise mechanism driving exclusion of T-cells from topographies enriched in DZ-related genes is elusive. It is reasonable that a convergence of different mechanisms is responsible for the observed dynamics, including the engendering of a DZ/LZ separation through chromatin compaction gradients, the overexpression of secreted factors with repulsive effects over T-cell subsets such as BMP7^58^ and CXCL12^64^, the DZ environment ruled by metabolically super-competitive cells sustained by FOXO1 signaling^65^, the tight control over genomic instability, inflammatory signaling and antigen presentation programs.

In essence, our exploration of the GC DZ spatial biology uncovers a complex interplay of replication-associated DNA damage response, chromatin compaction, and immune regulation. Our findings hint at a conserved transcriptional core enriched in DNA replication and damage response programs linked to replicative stress, as a potential hallmark of T-cell depleted tumor contextures and point to ATR inhibition as a candidate strategy to effectively revert these conditions. The study’s main limitations include the lack of a dynamic model to analyze changes in T-cell distribution and activation in GC DZ and DZ-like lymphomas within their natural environment. Additionally, while ATR targeting presents a promising new method to affect DZ-related molecular processes, it may unpredictably impact the natural dynamics of the GC reaction. The association of the described DZ spatial signature with molecular programs related to cell replication and DNA-damage response suggests its potential role as a negative regulator of T-cell infiltration in non-B lineage tumors as well, warranting further investigation.

## MATERIALS AND METHODS

### Murine models

*Aicda*^tm1(cre)Mnz/J^ (JAX:007770) and Wild Type C57BL6/J mice were obtained from Jackson Laboratory. Animals were regularly monitored by veterinary personnel throughout the duration of the experiments. Mice were checked at least three times a week for signs of illness and any reduction or impairment in motility. The experimental mice were followed until they reached 28–32 weeks of age. At this point they were euthanized to collect mesenteric lymph nodes for histopathological, immunolocalization and spatial transcriptomic analyses.

All animal procedures were approved by the *Animal Welfare Organization* (OPBA) of Palermo and the Italian Ministry of Health and carried out in accordance with Italian law (D.lgs 26/2014-authorization number 495/2020-PR).

### Human tissue samples

Formalin-fixed and paraffin-embedded (FFPE) samples of human tonsils with reactive follicular hyperplasia (20 cases) were selected from the archives of the Tumor Immunology Unit, University of Palermo, for in situ quantitative IHC and IF, mRNA ISH and PLA analyses.

One FFPE lymph node tissue sample involved by DLBCL was collected from the archives of the Pathology Unit of the University of Brescia for quantitative IF analyses and digital spatial profiling of microregions from DLBCL-infiltrated areas. The samples were collected and handled according to the Helsinki Declaration and the study was approved by the University of Palermo Ethical Review Board (approval numbers 09/2018 and 04/2023).

### Quantitative in situ hybridization and immunolocalization analyses

Single and multiplexed IHC and IF stainings, and in situ mRNA ISH were performed on FFPE human or murine tissue sections as previously described^66^. The detailed protocol and antibodies adopted are included in the Supplementary Methods. IHC-stained slides were digitalized using an Aperio CS2 digital slide scanner (Leica Microsystems) and IF-stained slides were analyzed and imaged under a Zeiss Axioscope-A1 equipped with widefield fluorescence module and Axiocam 503 Color camera (Zeiss). Quantitative analyses were performed using HALO image analysis software for cell segmentation and signal quantification (v3.2.1851.229, Indica Labs) as detailed *Spatial Analysis* paragraph of the Supplementary Methods.

### In situ Proximity Ligation assay (PLA)

The Proximity ligation assay (PLA) was conducted on FFPE sections from human tonsil samples using the NaveniBright HRP kit or NaveniFlex Tissue MR Red kit following manufacturer’s instructions (Navinci Diagnostics). The list of antibodies adopted for test and control PLA assays is included in the Supplementary Methods. Quantitative analysis of PLA signals has been performed through HALO image analysis software (v3.2.1851.229, Indica Labs) as detailed in the Supplementary Methods.

### In situ transcriptional analyses

We analyzed the transcriptional landscape of 10 DZ/LZ ROIs within morphologically normal FFPE tonsil GCs profiled by Nanostring Digital Spatial Profiling (NanoString, Seattle, WA). This analysis was performed on slides stained with CD271/NGFR (a marker for follicular dendritic cells to delineate the LZ) and CD20 (a B-cell marker), as detailed in our previous work^18^. The selected and segmented DZ and LZ ROIs were profiled for the expression of 1,824 curated genes from the Cancer Transcriptome Atlas panel (https://www.nanostring.com/products/geomx-digital-spatial-profiler/geomx-rna-assays/geomx-cancer-transcriptome-atlas/) using the GeoMx Digital Spatial Profiler) (NanoString, Seattle, WA). Additionally, 11 ROIs from a FFPE lymph node tissue sample, infiltrated by DLBCL, were selected based on staining with CD20 and CD3 (a T-cell marker) and profiled for the same curated gene panel. Detailed information on DSP data analysis is reported in the Statistical and bioinformatics analyses paragraph and in the Supplementary Methods.

Spatial transcriptomics analysis on mouse FFPE mesenteric lymph nodes was performed using the 10X Visium system (10X Genomics), following the manufacturer’s instructions. Detailed information on the library preparation, sequencing and data analysis of the Visium spatial transcriptomics experiment is provided in the Supplementary Methods.

### Computational pipelines to characterize the chromatin states of DZ and LZ cells

A series of computational pipelines were developed to perform cell type classification according to nuclear chrometric features, Random Forest classification to capture chrometric differences between LZ/DZ B-cells, analysis of the chromatin states of B-cells in the context of their distance to the LZ/DZ interface chromatin compaction states, correlation analysis of the chromatin condensation of B-cells and their distance to T-cells, correlation analysis of the chrometric states and the DZ signature of selected in situ transcriptionally-profiled microregions, and statistical hypothesis testing on chrometric features. All these pipelines, which have been applied to digital images of 15 manually-identified GCs from AID/CD3 IF and to 11 DSP-profiled DLBCL ROIs stained for CD20/CD3 are reported *in extenso* in the Supplementary Methods.

### DLBCL PDX RNAseq analyses

RNAseq data from primary DLBCL tumors and PDX in NSG (NOD.Cg-Prkdcscid Il2rgtm1Wjl/SzJ mice) were relative to GSE145043 (^67,68^). Data acquisition and analysis and patient-derived xenograft (PDX) establishment were approved and carried out in accordance with IRBs from the New York-Presbyterian Hospital, Weill Cornell Medicine, New York, and Ospedale San Giovanni Battista delle Molinette, Turin, Italy^67,68^. All animal procedures followed National Institutes of Health protocols and were approved by the Animal Institute Committee of the Weill Cornell Medical College.

PDX were established in female and male NSG mice by subcutaneous injection of primary human DLBCL cells in both flanks for several passages. PDX tissues extracted and profiled by RNA-sequencing at passages P1–2 (early) or P3–5 (advanced) were analyzed. Additional details are available in the Supplementary Methods.

### DLBCL cell lines culturing and in vitro ATRi treatment experiments

HT and SUDHL-5 cell lines were selected based on the high expression of the DZ spatial signature according to the 23Q2 DepMap gene expression dataset (https://depmap.org/portal/download/all/). HT and SUDHL-5 cells were cultured in RPMI media supplemented with 1% glutamine, 10% fetal bovine serum (FBS) and penicillin-streptomycin. Suspension cultures were maintained in flasks in 5% CO_2_, at 37°C. The cells were treated for 48 hours with increasing dosages of the clinical-grade ATR inhibitor Ceralasertib (AZD6738 S7693 Selleckchem, 1 or 2 μM) and DMSO was added at a similar concentration in the untreated control. Additional informations are provided in Supplementary Methods section. Lamin B1 staining for analysis of micronuclei formation, RNA extraction, qPCR, and RNAseq on ATRi and control (DMSO-treated) cells are detailed in the Supplementary Methods.

### Competitive migration assay in microfluidic devices

Microfluidic devices were fabricated in PDMS (polydilmethylloxane), a bio compatible silicon elastomer, as previously reported (^69^). The device allowed to visualize the preferential PBMC migration towards ATRi- or DMSO-treated HT and SUDHL-5 cells embedded in 3D hydrogels as shown in Figure 9N. Details on cell loading, labeling, and quantitative analysis of cell migration and interactions are reported in the Supplementary Methods.

### Statistical and bioinformatics analyses

The spatial DZ and LZ signatures were obtained by comparing the gene expression of paired human tonsil DZ and LZ GC ROIs (n=10) profiled by Nanostring Digital Spatial Profiling as previously reported^18^. Upregulated/downregulated genes were selected using the limma moderated statistic^70^ (BH adjusted p-values < 0.05). The Reactome Pathway library was used for pathway enrichment analysis (ReactomePA R package)^71^. Specific pathways were selected through the Nanostring Panel Pro tool^72^. The Euclidean distance and the Ward.D2 method were used for unsupervised clustering. The SpatialDecon algorithm^23^ was adopted to estimate cell fractions on DSP data, while the xCell algorithm^39^ was used to estimate selected immune and stromal cell type enrichment scores on bulk RNA-seq samples.

Further details on unsupervised hierarchical clustering, pathway and gene set enrichment analyses, DZ/LZ Single-cell RNAseq analysis, DLBCL gene expression datasets adopted, Immune and stromal deconvolution, Gene expression correlation analysis, survival analysis on DLBCL datasets and Visium spatial transcriptomics analysis, are detailed in the Supplementary Methods.

## Data Availability

All data generated in the present work have been made publicly available. The DSP data relative to 11 profiled DLBCL ROIs have been reported in Supplementary Table 8. The human and mouse bulk RNA-seq fastq files have been deposited in Sequence Read Archive (SRA) under accession codes PRJNA1082634 and PRJNA1083017, while the read counts have been reported in Supplementary Tables 17 and 18. The raw and processed data of Visium Spatial transcriptomics have been deposited in GEO under the accession code GSE260998. The DSP RNA-seq data profiled on tonsil GC DZ and LZ ROIs are publicly available18. The PDX RNAseq data are publicly available on GEO (GSE145043).
